# Supplementary material for: Divalent europium-doped near-infrared-emitting phosphor for light-emitting diodes
Source: Nat Commun. 2019 Nov 20;10:5267. doi: 10.1038/s41467-019-13293-0 (PMC6868216; doi:10.1038/s41467-019-13293-0)
Supplement: Supplementary file 1 — Supplementary Information [file 41467_2019_13293_MOESM1_ESM.pdf]

Supplementary Information for

Divalent Europium-Doped Near-Infrared-Emitting Phosphor for Light-Emitting Diodes

Qiao et al.

Supplementary Figures

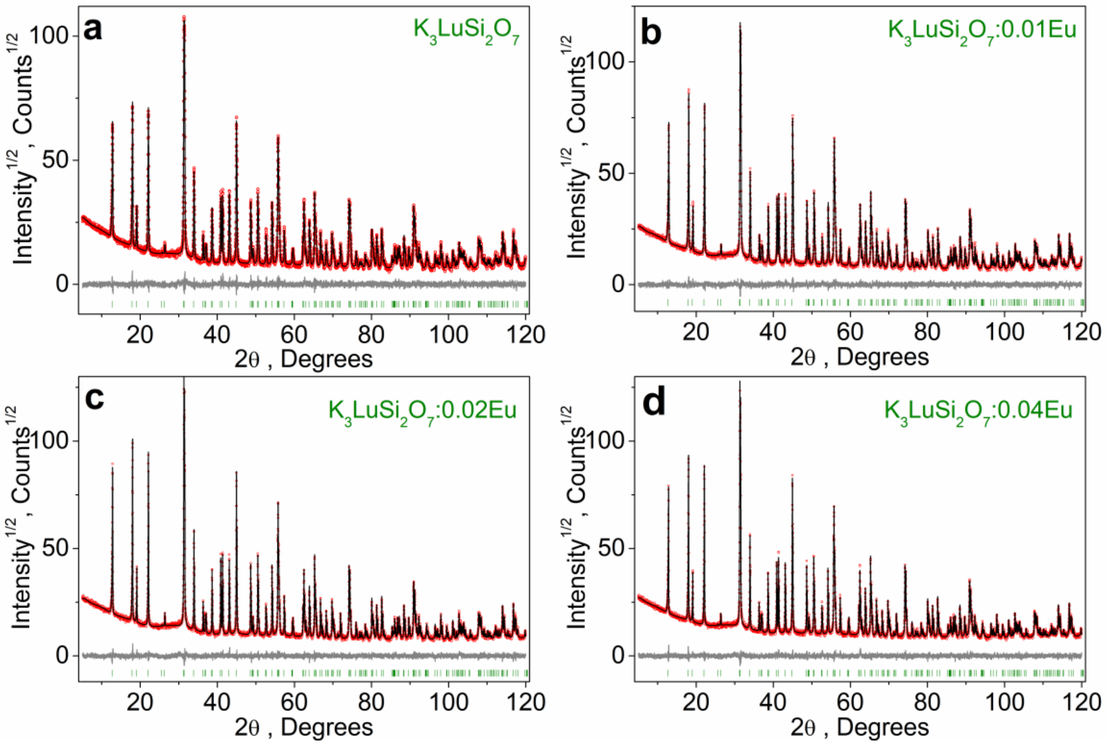

Supplementary Figure 1. XRD patterns. Difference Rietveld plot of $K_3LuSi_2O_7$:xEu for different Eu contents: **a** x = 0; **b** x = 0.01; **c** x = 0.02; **d** x = 0.04.

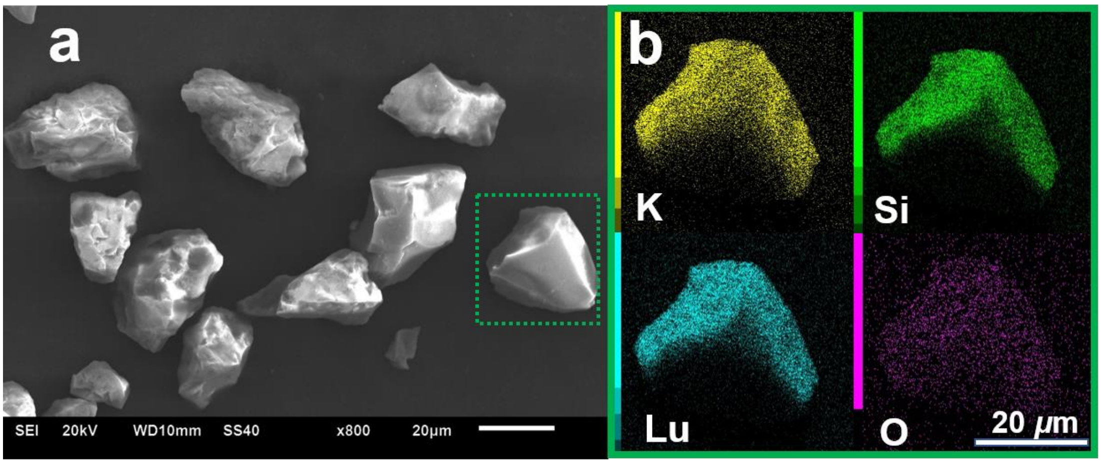

Supplementary Figure 2. SEM images. a SEM images of the $K_3LuSi_2O_7$:0.01Eu microcrystal particles. **b** Element mapping images of K, Lu, Si and O for the selected $K_3LuSi_2O_7$:0.01Eu particle.

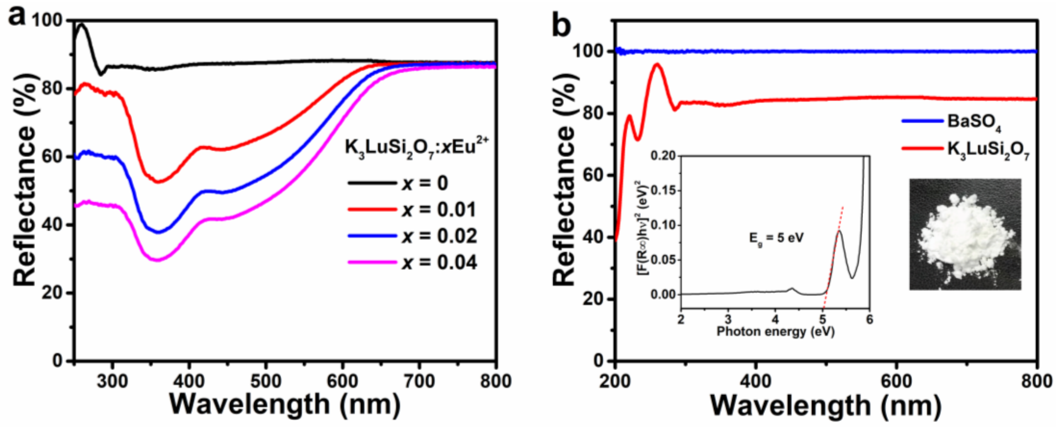

Supplementary Figure 3. Reflectance spectra and phosphor image. a Reflectance spectra of $K_3LuSi_2O_7$:$x$$Eu^{2+}$ with different doping concentrations. **b** Reflectance spectra of $BaSO_4$ and $K_3LuSi_2O_7$ host. The inset shows the photograph of $K_3LuSi_2O_7$ host.

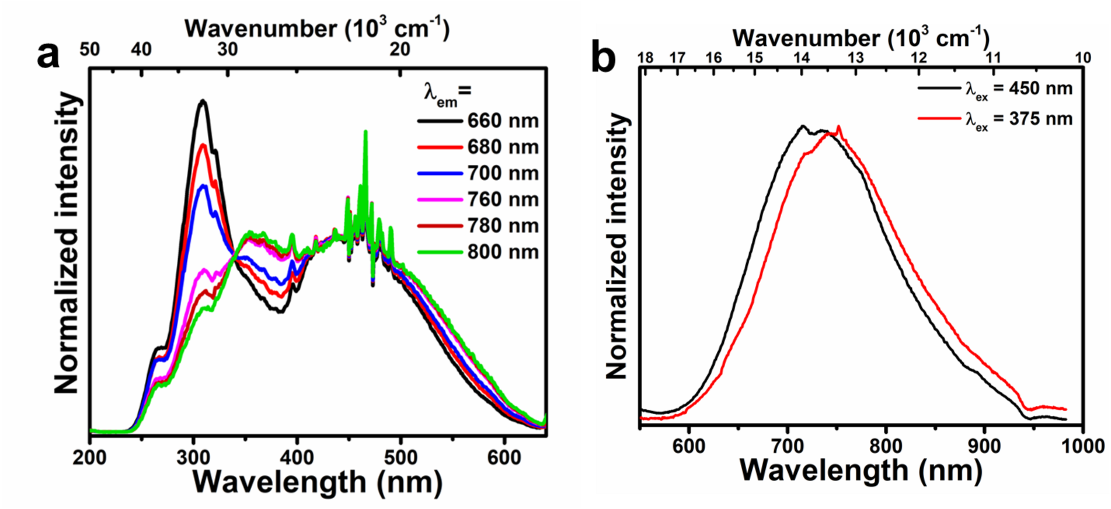

Supplementary Figure 4. PLE and PL spectra. a Normalized PLE spectra of $K_3LuSi_2O_7:Eu^{2+}$ monitored at different wavelength (The excitation intensity of 430 nm was used to normalize each PLE spectra). **b** Normalized PL spectra of $K_3LuSi_2O_7:Eu$ measured with fiber spectrophotometer under different excitation wavelength, 375 nm and 450 nm.

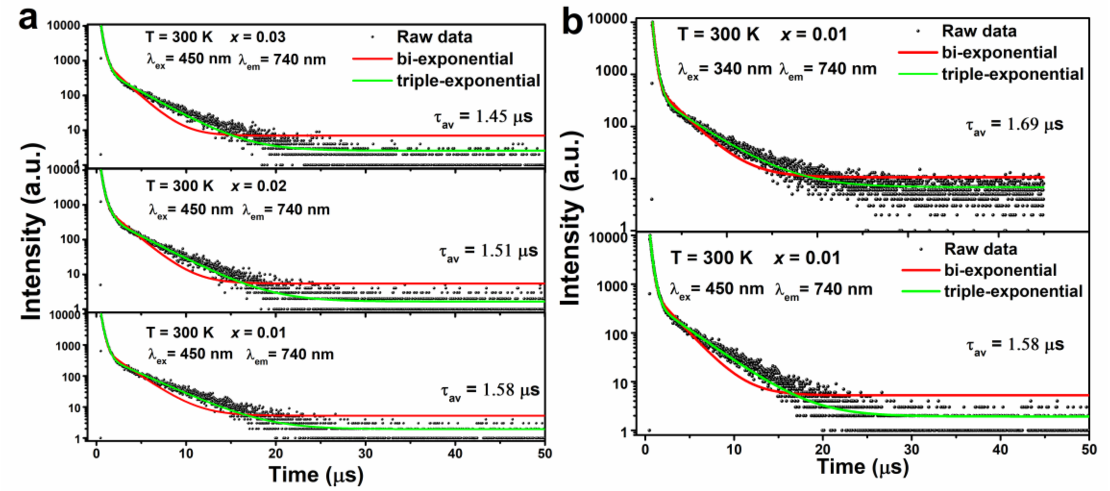

Supplementary Figure 5. Decay curves and the fitting difference. a Decay curves and fitting results of $K_3LuSi_2O_7$:*x*Eu (*x* = 0.01, 0.02, 0.03) measured at 300 K under 450 nm pulse laser diode excitation. **b** Decay curves and fitting results of $K_3LuSi_2O_7$:0.01Eu measured at 300 K under 450 nm and 340 nm pulse laser diodes excitation. (The decay curves were measured by FLS920 instrument equipped with 340 nm and 450 nm pulse laser diodes as the excitation source. The statistical photons are 10000. All the data were fitted by the FAST software attached with FLS920.)

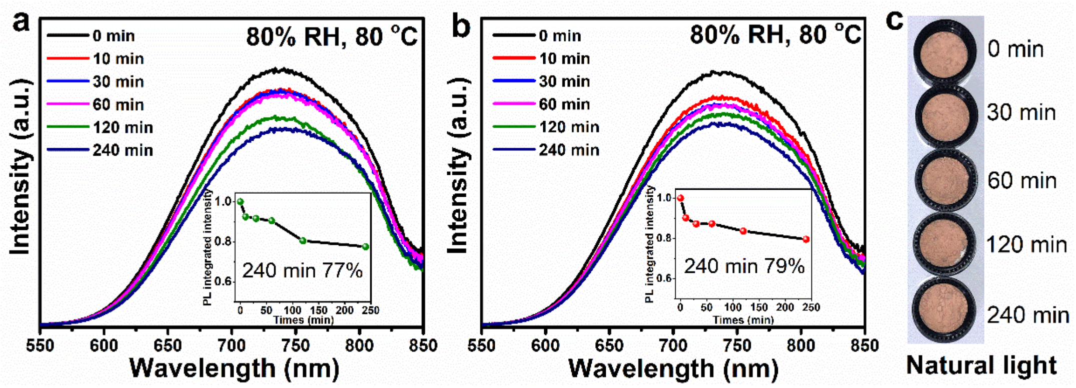

Supplementary Figure 6. Time dependent PL spectra and images. a, b The PL spectra of the pristine $K_3LuSi_2O_7$:Eu and the sample treated in degradation conditions at 80% relative humidity (RH), 80 °C for different time, respectively. The inset shows the dependence of normalized integrated PL intensities on the time. **c** Digital photographs of these samples under natural light.

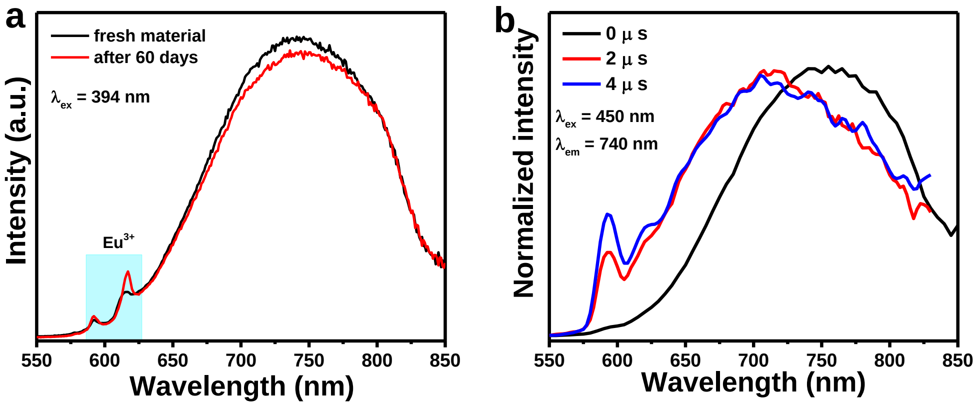

Supplementary Figure 7. PL and TRS curves. a The PL spectra of freshly prepared $K_3LuSi_2O_7$:0.01Eu and the samples exposed to the air for 60 days, under 394 nm excitation. **b** Normalized time-resolved luminescence spectra of $K_3LuSi_2O_7$:0.01Eu under 450 nm excitation.

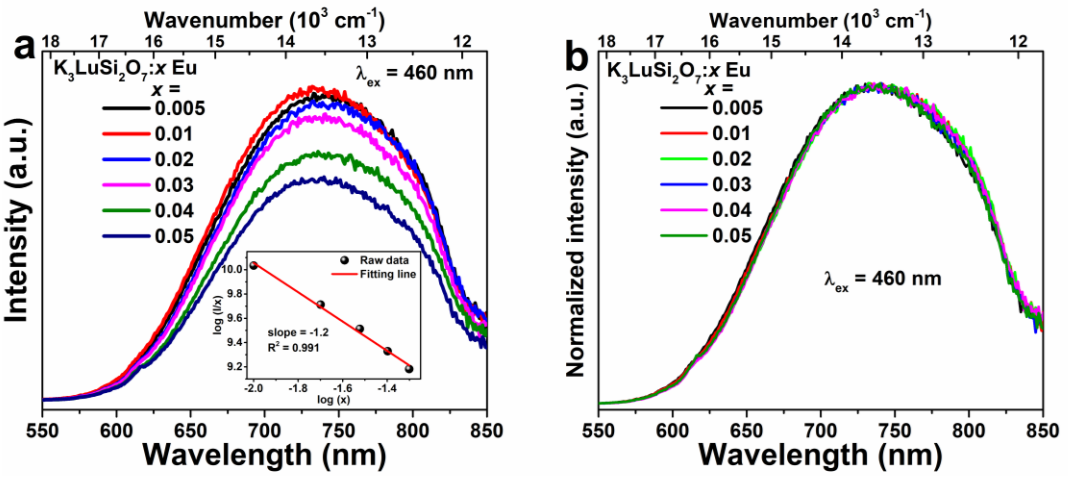

Supplementary Figure 8. PL spectra. a Unnormalized and **b** normalized emission spectra of $K_3LuSi_2O_7$:*x*Eu (*x* = 0.005-0.05) exciting at 460 nm.

Supplementary Tables

Supplementary Table 1. Main parameters of processing and refinement of the $K_3LuSi_2O_7$:*x*Eu samples

x	0	0.01	0.02	0.04
Sp. Gr.	$P6_3/mmc$	$P6_3/mmc$	$P6_3/mmc$	$P6_3/mmc$
a, Å	5.72186 (5)	5.72361 (7)	5.72398(3)	5.72462 (2)
c, Å	13.92097 (15)	13.9274 (2)	13.92958 (8)	13.93215 (7)
V, Å^3	394.707 (8)	395.131 (11)	395.244 (4)	395.405 (4)
Z	2	2	2	2
2θ-interval, °	5-120	5-120	5-120	5-120
R_{wp}, %	8.83	7.72	7.54	5.66
R_p, %	6.73	5.82	5.66	7.45
R_{exp}, %	6.27	6.01	5.91	5.75
χ^2	1.41	1.28	1.28	1.27
R_B, %	1.91	2.64	1.95	2.60

Supplementary Table 2. Fractional atomic coordinates and isotropic displacement parameters (Å^2) of $K_3LuSi_2O_7$:*x*Eu

Atom	*x*	*y*	*z*	B_{iso}	*Occ.*
			x = 0		
Lu	0	0	0	0.25 (4)	1
K1	1/3	2/3	0.09185 (15)	0.85 (5)	1
K2	0	0	1/4	1.20 (5)	1
Si	2/3	1/3	0.1311 (2)	0.23 (6)	1
O1	0.3566 (6)	0.1783 (3)	0.09676	0.72 (8)	1
O2	2/3	1/3	1/4	0.86 (17)	1
			x = 0.01		
Lu	0	0	0	0.43 (4)	1
K1	1/3	2/3	0.09233 (14)	1.13 (5)	1
K2	0	0	1/4	1.44 (5)	1
Si	2/3	1/3	0.13226 (19)	0.61 (6)	1
O1	0.3518 (5)	0.1759 (3)	0.09676	0.89 (8)	1
O2	2/3	1/3	1/4	1.94 (17)	1
			x = 0.02		
Lu	0	0	0	0.47 (4)	1
K1	1/3	2/3	0.09185 (13)	1.10 (5)	1
K2	0	0	1/4	1.43 (5)	1
Si	2/3	1/3	0.13196 (19)	0.66 (6)	1
O1	0.3529 (5)	0.1765 (2)	0.09676	0.75 (7)	1
O2	2/3	1/3	1/4	1.49 (16)	1
			x = 0.04		
Lu	0	0	0	0.35 (4)	1
K1	1/3	2/3	0.09223 (14)	1.05 (5)	1
K2	0	0	1/4	1.29 (5)	1
Si	2/3	1/3	0.1328 (2)	0.68 (6)	1
O1	0.3537 (5)	0.1768 (3)	0.09676	0.60 (8)	1
O2	2/3	1/3	1/4	1.71 (18)	1

Supplementary Table 3. Main bond lengths (Å) of $K_3LuSi_2O_7$:*x*Eu

	x = 0		
Lu—O1	2.224 (19)	Si—O1	1.609 (2)
K1—O1	2.864 (3)	Si—O2	1.655 (3)
K2—O1	2.77 (15)		
	x = 0.01		
(Lu/Eu)—O1	2.204 (15)	Si—O1	1.637 (2)
K1—O1	2.864 (2)	Si—O2	1.640 (3)
K2—O1	2.756 (12)		
	x = 0.02		
(Lu/Eu)—O1	2.208 (16)	Si—O1	1.631 (2)
K1—O1	2.864 (2)	Si—O2	1.644 (3)
K2—O1	2.756 (13)		
	x = 0.04		
(Lu/Eu)—O1	2.212 (15)	Si—O1	1.631 (2)
K1—O1	2.865 (2)	Si—O2	1.633 (3)
K2—O1	2.762 (12)		

Supplementary Table 4. The ionic radii of Eu^{2+}, K^{+}, Rb^{+}, Y^{3+} , Lu^{3+} in the different fold of coordination

Ion	Coordination number	Ionic radii (Å)
Lu^{3+}	6	0.86
Y^{3+}	6	0.90
K^{+}	6	1.37
K^{+}	9	1.55
Rb^{+}	6	1.52
Rb^{+}	9	1.63
Eu^{2+}	6	1.17
Eu^{2+}	9	1.30

Supplementary Table 5. Lists of the luminescent decay times (τ_1, τ_2, τ_3), fitting constants (A_1, A_2, A_3) average decay times (τ_{av}) and standard error of τ_{av} during the fitting of the decay curves of $K_3LuSi_2O_7$:Eu samples with different Eu^{2+} contents, which are measured at 80 K and 300 K under 450 nm and 340 nm pulse laser diodes excitation

λ_{ex}= 450 nm, λ_{em} = 740 nm, Temperature = 300 K								
x	A1	τ_1	A_2	τ_2	A_3	τ_3	τ_{av} (μs)	τ_{av} Error
0.01	7538.88	0.22	1557.41	0.62	328.31	4.01	1.58	0.04
0.02	7870.61	0.24	1569.44	0.73	382.01	3.74	1.51	0.03
0.03	7200.87	0.21	1664.63	0.69	353.89	3.61	1.45	0.03
λ_{ex}= 450 nm, λ_{em} = 740 nm, Temperature = 80 K								
0.01	1907.67	0.80	2519.20	1.55	312.02	5.35	2.28	0.03
λ_{ex}= 340 nm, λ_{em} = 740 nm, Temperature = 300 K								
0.01	8120.81	0.22	1381.83	0.63	324.79	4.26	1.69	0.04

Supplementary Table 6. Performance of prototype NIR-LED using the single $K_3LuSi_2O_7$:Eu phosphor

Current (mA)	Voltage (V)	Input power (mW)	Optical power (mW)
20	2.687	53.74	4.82
40	2.813	112.5	8.74
60	2.830	169.8	12.37
80	3.075	245.7	17.21
100	3.008	303.1	21.54
120	3.001	360.6	25.12

Supplementary Notes

Supplementary Note 1:

The body color of $K_3LuSi_2O_7$ host is white under natural light. The band gap is estimated by the following equation:[1]

$$\frac{[F(R_\infty)h\upsilon s]^2}{A} = h\nu - E_g \quad (1)$$

The value of the band gap *Eg* is about 5 eV.

Supplementary Note 2:

The average Eu-Eu distances (R_C) at the various doping levels are evaluated using the following equation:[2]

$$R_C = 2\left[\frac{3V}{4\pi X_C N}\right]^{1/3} \qquad (2)$$

where X_C is the doping concentration of activator ions; N is the number of cations which can be substituted by the dopant in per unit cell; and V is the volume of the unit cell. In the case of $K_3LuSi_2O_7$:Eu phosphors, Eu will occupy two K2 sites and one Lu sites, so, N = 3. The values of R_C are calculated to be 18.46, 14.65, 12.80, 11.63, 10.79 Å for x = 0.01, 0.02, 0.03, 0.04 and 0.05 samples, respectively. Thus, the concentration quenching is not triggered by the exchange interaction type due to the corresponding critical distance for exchange interaction is about 3-5 Å.

According to Dexter's theory, the type of electric multipolar interaction can be calculated by using the following formula:[3]

$$I/x = \mathrm{K}[1 + \beta(\mathrm{x})^{\theta/3}]^{-1} \qquad (3)$$

where x is the activator concentration, I is the emission intensity, K and β are constants for the same excitation condition for a given host lattice; θ = 3 stands for energy transfer among the nearest neighbor ions, while θ = 6, 8 and 10 stands for dipole-dipole, dipole-quadrupole and quadrupole-quadrupole interactions, respectively. The inset of Supplementary Figure 8a shows the dependence of log(I/x) versus log(x). The value of θ is calculated to be 3.6, close to θ = 3. It indicates that energy transfer among the nearest neighbor ions is the main concentration quenching mechanism in $K_3LuSi_2O_7:Eu^{2+}$ phosphors.

Supplementary Note 3:

Average decay times are calculated by the following question:

$$\tau_{av} = \frac{A_1\tau_1^2 + A_1\tau_2^2 + A_3\tau_3^2}{A_1\tau_1 + A_2\tau_2 + A_3\tau_3} \quad (4)$$

where A_1, A_2 and A_3 are the corresponding fitting constants; τ_1, τ_2 and $\tau_3$are lifetimes for the fast and slow components.

The relationship of decay times τ and emission wavelength λ can be described by following equation:[4, 5]

$$\Gamma = \frac{1}{\tau} \propto \frac{n}{\lambda^3}\left(\frac{n^2+2}{3}\right)^2 |< 5d|\mu|4f >|^2. \quad (5)$$

This equation predicts a longer decay time with longer emission wavelength. Thus, the decay time of the near infrared phosphor $K_3LuSi_2O_7$:Eu (~1.5 μs) are large than that of red phosphors (~1 μs).[6]

Supplementary References

1. Tauc, J., Grigorovici, R., Vancu, A. Optical properties and electronic structure of amorphous germanium. *Phys. Stat. Sol.* **15**, 627-637 (1966).
2. Blasse, G. Energy transfer in oxidic phosphors. *Philips Res. Rep.* **24**, 131-144 (1969).
3. Dexter, D. L. A Theory of Sensitized Luminescence in Solids. *J. Chem. Phys.* **21**, 836-850 (1953).
4. Duan, C. K., Reid, M. F. Local field effects on the radiative lifetimes of Ce^{3+} in different hosts. *Current Appl. Phys.* **6**, 348-350 (2006).
5. Dorenbos, P. Fundamental Limitations in the Performance of Ce^{3+}–, Pr^{3+}–, and Eu^{2+}–Activated Scintillators. *IEEE Trans. Nucl. Sci.* **57**, 1162-1167 (2010).
6. Qiao, J. *et al.* Site-Selective Occupancy of Eu^{2+} Toward Blue-Light-Excited Red Emission in a $Rb_3YSi_2O_7$:Eu Phosphor. *Angew. Chem. Int. Ed.* **58**, 11521-11526 (2019).
